# Ho! for Baltimore!

**Published:** 1895-04

**Authors:** 


HO! FOR BALTIMORE!—The attraction at Baltimore is unprecedented. There will be a very full attendance of the American Medical Association, and the sections and the general sessions will be held under one roof. The railroads have made arrangements to run special trains of vestibule cars, with dining-room coaches and sleepers, at the usual reduced rates, and will grant delegates and their families special excursions out of Baltimore at round trip rates; thus enabling one during the thirty days for which his ticket to Baltimore and return is good, to make a trip to New York or Philadelphia, and put in a month at the Polyclinics or Post Graduate schools and big hospitals; to run down the Potomac to Mt. Vernon and Old Point Comfort; there will be a special excursion to the Gettysburg battlefield, and a day spent on the grounds. At Baltimore, in addition to the splendid scientific entertainment prepared, the social features will be brilliant and pleasant. There will be a grand reception by the Johns-Hopkins Hospital, and another by the local profession, at one of the leading hotels. Texas delegates should time their departure so as to catch the "Big 4" train, which leaves St. Louis Sunday afternoon, May 5. That is the best route. Delegates from South and West Texas—Galveston, Laredo, etc.,—should take the I. & G. N. R. R. to Longview. From West and Central Texas, the T. & P. to Longview, the T. & P. from Longview to Texarkana, thence the Iron Mountain Route to St. Louis, thence over the Big 4 to Cincinnati, and the Chesapeake & Ohio thence to Washington. This latter takes them through the famous Cheat River country, famous for its lovely scenery, lovely at all times, but grand in its spring attire. Tickets will be on sale at all depots on the above lines, and agents will be pleased to give all desired information. Let every Texas doctor, who can possibly get off, make his arrangements to enjoy the "trip to Baltimore." (May 7th to 10th inclusive.)

